# Eye Movements Reveal Effects of Visual Content on Eye Guidance and Lexical Access during Reading

**DOI:** 10.1371/journal.pone.0041766

**Published:** 2012-08-08

**Authors:** Kevin B. Paterson, Victoria A. McGowan, Timothy R. Jordan

**Affiliations:** College of Medicine, Biological Sciences and Psychology, University of Leicester, Leicester, United Kingdom; George Mason University/Krasnow Institute for Advanced Study, United States of America

## Abstract

**Background:**

Normal reading requires eye guidance and activation of lexical representations so that words in text can be identified accurately. However, little is known about how the visual content of text supports eye guidance and lexical activation, and thereby enables normal reading to take place.

**Methods and Findings:**

To investigate this issue, we investigated eye movement performance when reading sentences displayed as normal and when the spatial frequency content of text was filtered to contain just one of 5 types of visual content: very coarse, coarse, medium, fine, and very fine. The effect of each type of visual content specifically on lexical activation was assessed using a target word of either high or low lexical frequency embedded in each sentence

**Results:**

No type of visual content produced normal eye movement performance but eye movement performance was closest to normal for medium and fine visual content. However, effects of lexical frequency emerged early in the eye movement record for coarse, medium, fine, and very fine visual content, and were observed in total reading times for target words for all types of visual content.

**Conclusion:**

These findings suggest that while the orchestration of multiple scales of visual content is required for normal eye-guidance during reading, a broad range of visual content can activate processes of word identification independently. Implications for understanding the role of visual content in reading are discussed.

## Introduction

Normal reading relies critically on moving the eyes along lines of text and activating lexical representations so that words can be identified accurately. Indeed, it is widely argued that the process of word identification is the engine that drives the forward movement of the eyes when reading [1]–[3], and abundant evidence shows that decisions about when to move the eyes are strongly influenced by how easily words can be identified (for a general review of this and other issues in eye movement research, see [4], [5]). However, little is yet known about what visual content present in text can support normal eye guidance and activate lexical representations during reading.

It is of particular importance for the present research that although words may appear to be composed only of letters, words are actually complex visual stimuli that contain an array of different types of visual content [6]–[10]. These range from coarse scales of visual content that may primarily be useful for determining the layout of text and the overall, size, shape, location, and orientation of letters and words, to more fine-scale visual content that may be of greater help in specifying individual letters and letter features (for further discussion, see [11]). This issue has been widely discussed in relation to the long-established view that the human visual system operates in the spatial frequency domain, supported by psychophysical and anatomical evidence that pathways in the human visual system are sensitive to spatial frequencies associated with different scales of visual content [12], [13]. Indeed, reduced sensitivity to the visual content produced by certain spatial frequencies has previously been associated both with dyslexia [14], [15] and poor reading ability in young adult readers [9], [10]. However, the visual content of text that can support eye guidance and activate lexical representations during normal reading remains to be determined.

A specific concern for the present research is the proposal that reading may require only some spatial frequencies to be processed, and therefore that text may be read normally using only part of its normal visual content (for discussions see [16]–[19]). An approach taken previously to investigate this issue compared reading rates obtained when text was displayed normally and when spatial frequencies were filtered so that the same text contained only some of its original visual content ([17], [18], [20], see also [9], [10]). The logic of this approach is straightforward. If readers require a particular type of visual content to read normally, normal reading performance will be unaffected when this visual content is present and impaired when this visual content is absent. For instance, Legge et al. [18] used a visual filter to remove certain medium and fine visual content from text so that only relatively coarse visual content remained. This text was presented in a scrolling display in which lines of text moved in a smooth sweeping motion from right to left so that participants could read each line without moving their eyes from one word to the next. Reading rates were calculated from the speed of presentation at which participants made only a small number of errors while reading each line of text aloud. The results showed that reading rates for filtered text were largely unaffected relative to reading rates for unfiltered (normal) text, and this led Legge et al. to propose that this coarse visual content is all that is required for reading. In a similar vein, Leat and Munger [20] filtered sentences into various bands of visual content and found participants read equally fluently when only coarse, medium, or fine visual content was present, leading Leat and Munger to argue that a range of different visual content can produce normal reading performance. More recently, Chung and Tjan [17] used a rapid serial visual presentation (RSVP) paradigm in which participants read aloud words presented sequentially at the same screen location and filtered into various types of visual content. Reading rates were calculated from the speed of presentation at which 80% of words were read correctly. Participants were able to read words across a wide range of different visual content equally quickly, and reading rates were reduced only when very coarse or very fine visual content was presented. Chung and Tjan suggest that normal fluent reading can be achieved using only part of the normal visual content of text, and this may extend across a range of different types of visual content. Although not making quite this claim, Patching and Jordan [9], [10] report similar findings from single-word recognition studies which showed that when only narrow bands of visual content were presented, words were recognized most accurately when only their coarse, medium, or fine visual content was displayed, and recognized least accurately when only their very fine or very coarse visual content was displayed.

In sum, prior research suggests that a wide range of visual content can support normal reading and word recognition, and some studies even suggest that normal reading uses only a narrow subset of the normal visual content of text. But this research has relied on measures such as overall reading speed and recognition accuracy to assess reading performance without analyses of eye movement behavior, and some studies have intentionally employed techniques in which eye movements are not required. Studies that assessed reading rates have also typically examined oral reading, and a considerable amount of evidence shows that, compared to normal silent reading, reading performance differs substantially when reading aloud, because readers must articulate each word as it is encountered and fixations typically remain in the same place for longer so that they do not get too far ahead of the word being spoken ([21]; see also [4], [5]). As a result, the findings from this prior research are not instructive about how the visual content of text supports normal processes of eye guidance and lexical activation during natural (silent) reading, and thereby enables normal reading to take place. Indeed, a wealth of research shows that eye movements during reading are not only necessary but are also remarkably informative about the processes determining when and where to move the eyes and the processes underlying lexical activation [4], [5] and this research has informed the development of sophisticated models of eye guidance during reading [1], [2], [22].

**Figure 1:**
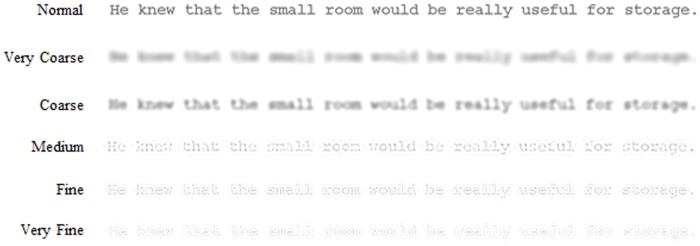
Examples of a sentence displayed as normal and containing only one type of visual content. (a) Very Coarse, (b) Coarse, (c) Medium, (d) Fine, (e) Very Fine. The appearance of each visual content shown in the figure is approximate due to restrictions in resolution and print medium.

Accordingly, to gain clearer insight into effects of visual content on eye guidance and lexical activation during reading, the present research compared the eye movement performance obtained when reading sentences displayed entirely as normal with that obtained when reading sentences in which spatial frequencies were filtered so that text was displayed in various bands of restricted visual content ranging from very fine to very coarse (see Figure 1). The influence of this sparse visual content on the general characteristics of eye guidance was assessed using established eye movement measures (namely, reading time, average fixation duration, number of fixations, number of regressions, and the amplitude of progressive saccades). In line with previous research [9], [10], [17], [18], [20], if normal eye movement performance requires certain visual content, normal eye movement performance will be disrupted when text lacks this visual content.

However, these displays and measures of eye movement performance also enabled the influence of visual content on lexical processing during reading to be investigated. It is well-established that the duration of fixations on words during reading is highly sensitive to the lexical frequency of the fixated word, and that readers typically spend longer fixating words of lower written frequency [23]–[31]. This influence is often attributed to the greater familiarity of higher frequency words facilitating an early stage of word identification associated with lexical access [32], [33]. Moreover, recent findings suggest that lexical frequency effects appear even when text is altered to simulate optical aberrations that distort the physical form of letters in words [34], suggesting that lexical frequency effects provide a robust measure of lexical access. Accordingly, the effects of each type of visual content on lexical activation were investigated in the present experiment by assessing eye movements for target words that were of either high or low written frequency and that were embedded in each sentence.

Prior research suggests that text presented in a broad range of restricted visual content can be read relatively normally [17], [18], [20], but the effect on the process of lexical identification during reading remains to be determined. Indeed, if only certain types of visual content provide access to lexical representations during reading (presumably including visual content used in prior research), lexical frequency effects should be apparent in fixation durations for target words that contain this visual content, and should be absent from target words that do not. Prior research has also shown that fixation durations are sensitive to the difficulty with which words can be identified and that, when word identification is more difficult, processing lower frequency words is impaired disproportionately and a larger effect of lexical frequency is observed [26], [29]. Consequently, if some visual content is better than others at supporting lexical access, this difference in the effectiveness of visual content should also be revealed by the effects of lexical frequency on fixation durations for target words. Indeed, if the removal of certain spatial frequency content is particularly disruptive, additional processing may be required to activate lexical representations for target words and, if this were to happen, the appearance of lexical frequency effects in the eye movement record may be delayed. However, as measures of fixation duration are highly sensitive to the timing of lexical influences on word identification [5], fixation durations when visual content is restricted are well-suited to revealing whether effects of lexical frequency are observed early or late during processing.

Analyses of eye movements for specific target words in sentences also enabled an assessment of the incidence which these words were skipped during reading, and the location of initial fixations (often called the initial landing positions) within these words. The frequency of word skipping has been shown to be informative about the processing of words in readers' right parafovea, as words are more likely to be skipped if they can be identified in parafoveal vision [35], [36]. In addition, it is well-established that there is a systematic tendency for readers' initial fixations to land just to the left of the middle of words during normal reading [37], and so initial landing positions in words are informative about the accuracy of saccade targeting during reading. These analyses provide further insights into the effects of visual content on word processing and eye guidance during reading. Accordingly, if only certain visual content produces normal eye guidance, the frequency of word-skipping and the location of landing positions should not differ for text displayed in this visual content compared to text displayed normally. By comparison, if restricting the visual content present in text impairs the parafoveal processing of target words, this will lower rates of word-skipping, and if this impairs accuracy of saccade targeting, patterns of initial landing positions in target words will differ for sentences displayed in restricted visual content compared to sentences shown normally.

## Results

Each sentence was followed by a question that assessed readers' comprehension. Accuracy in responding to these comprehension questions was generally high, but lower for very coarse visual content than for any other display condition, *F*(3.16,91.77) = 77.43, *p*<.001, η_p_^2^ = .73 (see Table 1). This indicated that although readers comprehended text normally when text contained a broad range of restricted visual content (ranging from coarse to very fine), normal text comprehension was substantially impaired when text contained only very coarse visual content.

**Table 1:** Eye Movement Measures for Each Display Condition.*

	Display Condition					
	Normal	Very Coarse	Coarse	Medium	Fine	Very Fine
Comprehension Accuracy (%)	96 (.8)	67 (2.5)	92 (2.1)	97 (.7)	94 (1.4)	95 (1.5)
Sentence Reading Time (ms)	2861 (184)	8157 (738)	5657 (363)	3378 (203)	3629 (289)	4239 (318)
Average Fixation Duration (ms)	228 (4.7)	365 (7.6)	300 (5.7)	247 (4.7)	257 (9.4)	278 (11.8)
Total Number of Fixations	10.6 (.63)	19.7 (1.75)	16.5 (.94)	11.7 (.61)	12.0 (.59)	13.1 (.59)
Number of Regressions	2.5 (.13)	6.8 (.32)	4.5 (.20)	2.7 (.13)	2.6 (.11)	2.8 (.13)
Forward Saccade Length (Characters)	9.5 (.3)	8.7 (.5)	8.0 (.2)	8.8 (.3)	8.4 (.3)	7.7 (.2)

* Standard Errors are provided in parentheses.

### Global Measures

A range of global eye movement measures was computed to assess the general characteristics of eye movement performance [4], [5]; these were reading times, average fixation duration, number of fixations, length of progressive saccades, and number of regressions (see Table 1). The effects of display condition (normal plus 5 types of visual content) on these measures were analyzed using a one-way analysis of variance (ANOVA) computing error variance over participants (*F*_1_) and sentences (*F*_2_), and using the Greenhouse-Geisser correction where appropriate. We also report partial eta-squared (η_p_^2^) as a measure of effect size based on the proportion of variance in the dependent variable that is attributable to our experimental manipulation. Pairwise comparisons were performed using a Bonferroni-corrected Tukey test (adjusted *p*<.05 for all significant effects). This involved computing pair-wise comparisons of the values for each display condition (i.e., a total of 15 comparisons for each eye movement measure).

Relative to normal displays, every restricted visual content slowed reading times, lengthened average fixation duration, and produced more fixations (reading times, *F*_1_(1.28,29.37) = 36.66, *p*<.001, η_p_^2^ = .61, *F*_2_(3.36,399.26) = 192.27, *p*<.001, η_p_^2^ = .62; average fixation duration, *F*_1_(1.70,38.98) = 97.55, *p*<.001, η_p_^2^ = .81, *F*_2_(2.18,378.08) = 374.99, p<.001, η_p_^2^ = .76; number of fixations, *F*_1_(1.22,28.14) = 23.22, p<.001, η_p_^2^ = .50, *F*_2_(3.89,462.78) = 79.56, *p*<.001, η_p_^2^ = .40). These impairments to normal eye movement performance were greatest for very coarse visual content, smaller but still substantial for coarse visual content, smaller still for very fine visual content, and smallest of all for medium and fine visual content. Display condition also affected the length of progressive saccades, *F*_1_(1.68,38.57) = 9.18, *p*<.01, η_p_^2^ = .29, and *F*_2_(3.28,193.46) = 20.27, *p*<.001, η_p_^2^ = .26, which were shorter than normal for every restricted visual content. Progressive saccades were shortest for very fine visual content, longer for fine and coarse visual content, and longer still for medium and very coarse visual content. Finally, more regressions were made for coarse and very coarse visual content compared to normal displays, *F*_1_(1.33,30.52) = 22.87, *p*<.01, η_p_^2^ = .50, *F*_2_(2.81,165.65) = 257.54, *p*<.001, η_p_^2^ = .73, but regression rates did not differ from normal for medium, fine, or very fine visual content.

These global eye movement measures show that text displayed in any restricted band of visual content was more difficult to read than text displayed normally, indicating that no single type of visual content could support normal reading. Normal reading performance was nevertheless generally disrupted least by medium and fine visual content and disrupted most by coarse, very coarse, and very fine visual content. Therefore, although most types of visual content supported relatively good reading performance, information supplied by medium and fine visual content provided the most support. Moreover, the high levels of comprehension accuracy for text displays ranging from coarse to very fine showed that this broad range of visual content supports normal processes of comprehension. By contrast, normal processes of reading and comprehension appeared to be substantially disrupted when text was displayed in very coarse visual content, as reading performance was much poorer, and comprehension accuracy was much lower, in this display condition.

### Target Word Measures

The specific effects of visual content on lexical access during reading were assessed using a range of local eye movement measures for the target words of high or low lexical frequency embedded in each sentence [4], [5]. These were first fixation duration (the duration of the first fixation on a target word), gaze duration (the summed duration of all fixations on a target word before a saccade is made away from the word), total reading time (the summed duration of all fixations made on a target word, including re-fixations), number of first-pass fixations (the total number of fixations made on a word when it is first encountered), and the percentage probability of a first-pass regression from the target word back to an earlier location in the sentence. First fixation duration and gaze duration provide measures of early word processing, but total reading time includes time spent re-fixating a word after the first-pass processing of the word has been terminated and so effects that emerge in this measure are considered to reflect later stages of word processing. First-pass fixations are informative about how many fixations are required to process a word, and the percentage probabilities of first-pass regressions are informative about disruption of the normal left-to-right processing of text. In addition to these measures, we also examined the locations of initial fixations in target words (the initial landing positions) as these are informative about the accuracy of saccade targeting, and the percentage probabilities of skipping the target word as these are informative about the likelihood of readers fixating a target word during first-pass processing of text.

Prior to analyzing the eye movement data, fixations shorter than 80 ms or longer than 1200 ms were deleted (accounting for just 2.9% of the data). Data for each measure were then analyzed by performing a 6 (display condition)×2 (target word frequency) ANOVA, computing error variance over participants (*F*_1_) and stimuli (*F*_2_) and using the Greenhouse-Geisser correction where appropriate. Pair-wise comparisons were performed using a Bonferroni-corrected Tukey test (adjusted *p*<.05 for all significant effects). This involved computing pair-wise comparisons of the values for each display condition when assessing the effects of display condition on eye movement behavior (i.e., a total of 15 comparisons for each eye movement measure), and computing pair-wise comparisons of values for words with a high and low lexical frequency when assessing effects of lexical frequency for each display condition (i.e., a total of 6 comparison for each eye movement measure). Mean eye movement measures for high and low frequency target words in each display condition are shown in Table 2.

**Table 2:** Eye Movement Measures for High and Low Frequency Target Words for Each Display Condition.*

	Display Condition											
	Normal		Very Coarse		Coarse		Medium		Fine		Very Fine	
	Lexical Frequency											
	High	Low	High	Low	High	Low	High	Low	High	Low	High	Low
First Fixation Duration (ms)	204 (6)	224 (8)	353 (16)	357 (17)	296 (10)	327 (17)	227 (8)	263 (9)	235 (8)	277 (10)	259 (7)	294 (14)
LFE	20		4		31		36		37		35	
Gaze Duration (ms)	223 (10)	252 (10)	490 (28)	526 (39)	393 (23)	550 (46)	253 (9)	320 (17)	260 (11)	336 (15)	300 (14)	397 (33)
LFE	29		36		157		67		76		97	
Total Reading Time (ms)	619 (36)	724 (37)	1539 (165)	1731 (187)	1054 (71)	1609 (156)	721 (57)	905 (63)	724 (44)	888 (82)	907 (127)	1061 (101)
LFE	105		192		555		184		164		154	
Total Number of First-Pass Fixations	1.11 (.03)	1.17 (.02)	1.46 (.06)	1.46 (.07)	1.32 (.05)	1.66 (.08)	1.15 (.03)	1.26 (.05)	1.11 (.02)	1.33 (.08)	1.30 (.11)	1.40 (.08)
LFE	0.06		0		0.34		0.11		0.22		0.10	
First-Pass Regressions (%)	7.4 (2.6)	12.1 (2.7)	18.9 (3.6)	25.5 (4.5)	11.7 (2.4)	15.4 (2.5)	10.3 (2.2)	10.9 (2.5)	8.9 (1.8)	7.5 (2.3)	7.7 (2.2)	6.1 (1.4)
LFE	4.7		6.6		5.1		0.6		−1.4		−1.6	
Skipping (%)	20.0 (4.0)	7.9 (1.9)	22.1 (5.0)	18.8 (4.1)	6.3 (1.6)	2.5 (1.1)	9.2 (2.0)	7.1 (1.8)	6.7 (2.4)	7.9 (1.7)	4.2 (1.3)	4.6 (1.3)
LFE	−12.1		−3.3		−3.8		−2.1		1.2		0.4	
Landing Position (Characters)	2.4 (.1)	2.2 (.1)	1.9 (.1)	1.9 (.1)	2.2 (.1)	1.8 (.1)	2.5 (.1)	2.2 (.1)	2.3 (.1)	2.2 (.1)	2.4 (.1)	2.1 (.1)
LFE	.2		.0		.4		.3		.1		.3	

* LFE = Lexical Frequency Effect. Standard Errors are provided in parentheses.

A main effect of display condition was obtained for all fixation duration measures (first fixation duration, *F*_1_(3.25,74.76) = 37.69, *p*<.001, η_p_^2^ = .62, *F*_2_(3.32,195.67) = 73.59, *p*<.001, η_p_^2^ = .56; gaze duration, *F*_1_(2.56,58.79) = 34.84, *p*<.001, η_p_^2^ = .60, *F*_2_(2.94,173.46) = 58.08, *p*<.001, η_p_^2^ = .50; total reading time, *F*_1_(1.79,41.22) = 21.76, *p*<.001, η_p_^2^ = .49, *F*_2_(2.89,170.33) = 102.85, *p*<.001, η_p_^2^ = .64). First fixation durations for target words were shortest for normal displays, equally longer for medium and fine visual content, and increasingly longer for very fine, coarse, and very coarse visual content, respectively (*p*s<.05). The same pattern of effects was obtained for gaze durations except that gaze durations did not differ between coarse and very coarse visual content (*p*s<.05). Total reading times were equally short for normal displays and medium, fine, and very fine visual content, but longer for coarse and very coarse visual content (*p*s<.05).

Fixation durations also produced effects of target word frequency (first fixation duration, *F*_1_(1,23) = 26.40, *p*<.001, η_p_^2^ = .53, and *F*_2_(1,59) = 34.45, *p*<.001, η_p_^2^ = .37; gaze duration, *F*_1_(1,23) = 42.69, *p*<.001, η_p_^2^ = .65, *F*_2_(1,59) = 47.49, *p*<.001, η_p_^2^ = .45; total reading time, *F*_1_(1,23) = 58.01, *p*<.001, η_p_^2^ = .72, *F*_2_(1, 59) = 31.62, *p*<.001, η_p_^2^ = .35) due to longer fixation durations for lower frequency words than for higher frequency words. The interaction of display condition and target word frequency was not significant for first fixation durations (*F*s<1.8) but was significant for gaze durations, *F*_1_(2.75,63.24) = 4.47, *p*<.01, η_p_^2^ = .16, *F*_2_(3.19,188.45) = 3.14, *p*<.01, η_p_^2^ = .05, and total reading times, *F*_1_(3.33,76.51) = 6.19, *p*<.001, η_p_^2^ = .21, *F*_2_(2.93,172.57) = 4.26, *p*<.01, η_p_^2^ = .07. Effects of lexical frequency in gaze durations were significant for target words shown in normal, coarse, medium, fine, and very fine display conditions (*p*<.05), but not in the very coarse display condition (*p*>.12). However, effects of lexical frequency were obtained in total reading times for all display conditions, including very coarse (*p*s<.05).

Further analyses compared the size of the lexical frequency effects obtained for each display condition and showed no differences in the size of the effect for first fixation durations but did show differences in the size of the lexical frequency effect for gaze durations, where frequency effects were larger than normal for coarse, medium, fine, and very fine displays (*p*s<.05). Differences in the size of the lexical frequency effect were also observed for total reading times, where the large frequency effect for very coarse displays was greater than the frequency effects observed for all other display conditions (*p*s<.05). Moreover, as the lexical frequency effect for very coarse visual content emerged only in total reading times, it appears that effects of lexical frequency were delayed for this visual content, probably because very coarse visual content was more difficult to identify than words shown normally and additional processing was required for lexical access.

First-pass fixations produced an effect of display condition, *F*_1_(2.78,63.92) = 14.43, *p*<.001, η_p_^2^ = .39, *F*_2_(3.23,190.72) = 36.13, *p*<.001, η_p_^2^ = .38. The number of fixations on target words did not differ between normal and fine displays, but more fixations were made for medium displays, and most fixations were made for coarse, very coarse, and very fine displays (all *p*s<.05). First-pass fixations also showed an effect of target word frequency, *F*_1_(1,23) = 32.87, *p*<.001, η_p_^2^ = .59, *F*_2_(1,59) = 36.13, *p*<.001, η_p_^2^ = .38, due to more fixations for lower frequency words than for higher frequency words. There was also a significant interaction, *F*_1_(3.36,77.30) = 5.62, *p*<.001, η_p_^2^ = .20, *F*_2_(3.55,209.69) = 3.58, *p*<.01, η_p_^2^ = .06, which was due to larger lexical frequency effects for coarse and fine displays than for any other display condition (*p*s<.05). First-pass regressions also produced an effect of display condition, *F*_1_ (1.52,31.93) = 10.73, *p*<.001, η_p_^2^ = .34, *F*_2_(1.70,86.81) = 21.87, *p*<.001, η_p_^2^ = .30, due to more regressions for target words in coarse and very coarse displays than in any other display condition (all *p*s<.05). Skipping produced a main effect of display condition, *F*_1_(1.72,39.51) = 8.03, *p*<.001, h_p_^2^ = .26, *F*_2_(3.06,180.37) = 23.18, *p*<.001, h_p_^2^ = .28, due to lower skipping rates than normal, and therefore greater likelihood of fixating target words during first-pass processing, for all restricted visual content except very coarse (*p*s<.05). Skipping also produced a main effect of lexical frequency, *F*_1_(1,23) = 12.10, *p*<.01, h_p_^2^ = .35, *F*_2_(1,59) = 6.69, *p*<.05, h_p_^2^ = .10, which was qualified by an interaction with display condition, *F*_1_(3.41,78.46) = 3.71, *p*<.01, h_p_^2^ = .14, *F*_2_(4.20,247.57) = 2.60, *p*<.05, h_p_^2^ = .04. Higher frequency words were skipped more often than lower frequency words in normal and coarse displays (*p*s<.05) but no frequency effects were observed in skipping rates for very coarse, medium, fine, or very fine displays (*p*s>.20).

To examine effects of visual content on initial landings positions in words, we calculated the mean landing position (in characters) from the left boundary of target words (see Table 2). Initial landing positions produced a main effect of lexical frequency, *F*_1_(1,23) = 17.45, *p*<.001, η_p_^2^ = .43, *F*_2_(1,59) = 16.73, *p*<.001, η_p_^2^ = .22, due to fixations landing closer to the beginnings of target words that were of lower than higher frequency. Initial landing positions also differed across display conditions, *F*_1_(3.25,74.84) = 5.65, *p*<.01, η_p_^2^ = .20, *F*_2_(5,295) = 8.16, *p*<.001, η_p_^2^ = .12. For words presented normally, initial landing positions were on average 43% in from the left boundary of words (i.e., between characters 2 and 3 of words, which were a mean 5.32 characters long) and this replicated the standard finding that the preferred viewing position is a little to the left of a word's center [37]. Initial landing positions for normal displays did not differ for target words in medium, fine, or very fine displays (*p*s>.90), but were slightly (less than half a character) closer to the left boundary of target words in coarse and very coarse displays (*p*s<.05). There was no interaction of display condition and lexical frequency (*F*s<1.9).

The pattern of eye movements obtained for target words was broadly similar to that obtained for global eye movement measures, and showed generally that reading was more difficult when visual content was restricted compared to when sentences were presented normally. As with the global measures, fixation times for target words showed that normal reading performance was disrupted least by fine and medium visual content, disrupted more by coarse and very fine visual content, and disrupted most by very coarse visual content. Lexical frequency effects were obtained for fixation durations and total reading time for all visual content, and for gaze durations for coarse, medium, fine, and very fine visual content (as well as for text displayed normally), revealing that a broad range of visual content can support lexical access. Moreover, the lexical frequency effects produced for fixation durations and total reading time by a wide range of restricted visual content largely resembled the frequency effects obtained for normal displays, suggesting that the relative activation of high and low frequency lexical entries produced by normal text is supported by a range of visual content when reading. However, some lexical frequency effects (gaze durations) were larger than those produced by normal displays, suggesting that some aspects of word identification during reading were more difficult when visual content was restricted. Indeed, while lexical frequency effects were obtained for very coarse visual content, these appeared later in the eye movement record, in total reading times for target words, most likely because readers had difficulty in identifying words in this display condition. The size of this effect was also larger than that for normal displays, but smaller than that for coarse visual content. Based on the more general pattern of results we have observed, it seemed likely that word identification would be more greatly impaired by very coarse than coarse visual content, and therefore that very coarse visual content would produce larger effects of lexical frequency. However, the apparently smaller effect of lexical frequency for very coarse visual content likely occurred because the difficulty in identifying words in this display condition disrupted normal lexical frequency effects. The effects of restricted visual content on skipping rates suggest that removing all but the coarser visual content in text interfered with parafoveal word processing, since all but very coarse visual content produced reduced rates of word skipping compared to normal displays, and only coarse visual content produced an effect of word frequency (with lower rates of word skipping for lower frequency words) similar to that obtained for normal displays. However, the increased regressions and more leftwards landing positions for target words in coarse and very coarse visual content compared to normal displays indicate that coarse and very coarse visual content disrupted other aspects of normal eye movement control.

## Discussion

Measures of eye movement performance showed that restricting the visual content present in text produced longer reading times, longer average fixation durations, more fixations, and shorter progressive saccades compared to text shown as normal. Therefore, and in contrast with previous research that used measures of reading rates to assess effects of visual content on reading [17], [18], [20], these findings show that text is more difficult to read when it contains only this restricted visual content, and that no one type of visual content produces entirely normal reading performance. This crucial difference from previous findings is likely to be a consequence of the paradigms used to assess reading performance. In particular, previous research not only did not assess eye movement behavior but selected paradigms that eliminated the need for saccadic eye movements during reading and relied on measures of performance when text was read aloud. Unfortunately, saccadic eye movements are integral to normal reading, and abundant evidence shows that disruption to the normal planning of saccades can substantially affect reading performance [4], [5]. In addition, compared to normal silent reading, reading performance differs substantially when reading aloud. Consequently, previous research that used paradigms that distort or eliminate the need for eye movements is unlikely to provide a full and accurate assessment of normal reading performance. In contrast, eye movement measures that are highly informative about silent reading [4], [5] show that normal reading performance is not supported by any one type of restricted visual content.

It was also apparent that the various types of visual content used in our study differed in their ability to support reading. Eye movement performance was closest to normal for medium and fine visual content, which both produced faster reading times and fewer and shorter fixations than all other types of restricted visual content, and a similar rate of regressive saccades as text displayed normally. A similar advantage for medium and fine visual content in prior research [9], [10], [17] has been attributed to the importance of information about individual letters and letter features that facilitate word recognition and a similar benefit may have occurred for word identification in the present experiment. In a similar vein, reading times were slowest, and reading required more and longer fixations, for text in coarse or very coarse display conditions, suggesting that reading was difficult when this more detailed information was lacking. Indeed, the increased incidence of regressions for coarse and very coarse visual content compared to text displayed as normal suggest that difficulty in identifying words when fine-scale content is lacking impaired the normal left-to-right to right processing of text. This reading difficulty was particularly pronounced for very coarse visual content, and whereas the other types of visual content in this study were able to support good reading performance, and produced comprehension at levels similar to those obtained for text presented normally, it seems normal reading performance was disrupted substantially and, as a result, comprehension was particularly poor when only the very coarse visual content of text was displayed.

Other differences are also of interest. Very fine visual content produced faster reading times and fewer and shorter fixations compared to either coarse or very coarse visual content. Consequently, even though very fine visual content lacks the beneficial influence observed for medium and fine visual content, very fine visual content allowed text to be processed more efficiently than more coarse visual content, most likely because very fine visual content provided detailed information that was of particular help in establishing the identities of individual letters in words. However, text displayed in very fine visual content also received shorter progressive saccades compared to every other restricted visual content (including coarse and very coarse visual content), indicating that readers had difficulty in programming saccadic eye movements when text contained only very fine-scale information. Consequently, the advantage very fine visual content showed over coarser visual content in measures of reading time and fixation is offset by the difficulty it causes saccade planning. Accordingly, the indication from the present research is that no one type of visual content provides all the information required for all the components of natural reading, and therefore that a broad array of different visual content is required. Indeed, the present findings offer support for the view that natural reading requires information from a range of spatial scales, so that different visual content may contribute individually to the reading process but that normal reading involves the appropriate orchestration of these individual inputs [6]–[10], [17], [38], [39].

Measures of eye movement performance also enabled the influence of visual content on lexical processing during reading to be investigated more closely by examining performance for target words of high and low lexical frequency. Substantial previous evidence has shown that lexical frequency affects the duration of fixations on words, and that words of higher lexical frequency usually receive shorter fixations [23]–[31]. This lexical frequency effect is widely attributed to the facilitation of lexical access due to readers' greater experience of higher frequency words [32], [33]. Consequently, the finding that lexical frequency effects are produced by various types of restricted visual content provides the crucial demonstration that a broad range of visual content can produce lexical access independently during reading. As this effect emerged early in the eye movement record for various types of visual content (in gaze durations for all but very coarse visual content), the indication is that a broad range of visual content can initiate lexical access from early on in word recognition. Indeed, only very coarse visual content produced effects of lexical frequency that emerged late in the eye movement record (in total reading times for words), suggesting that lexical access was considerably slower and more difficult for this visual content, and that additional processing was required to activate lexical representations. Notably, the lexical frequency effects apparent for first fixation durations and total reading time when text was shown normally were similar for many types of restricted visual content, suggesting that the relative efficiency with which lexical entries of different frequencies of occurrence are normally activated is supported by a range of visual content when reading. However, gaze durations produced lexical frequency effects larger than those for normal displays, and larger effects of lexical frequency are associated with difficulty in identifying words [26], [29]. Consequently, it seems that while a broad range of visual content can produce lexical access, words are more difficult to identify when text lacks its normal rich complement of visual content. Nevertheless, in line with the findings from other global and local eye movement measures, the frequency-driven effects of visual content on lexical access suggests that a broad array of visual content may contribute independently to reading [6], [9], [10], [17], [38], [39].

Various proposals exist to explain how different types of visual content might support word recognition. For instance, because very coarse and coarse visual content may be processed ahead of other visual content, coarse-scale visual cues may normally provide the initial input for lexical access and this input is then normally augmented by finer-scale input (for discussions, see [6]–[10], [17], [38], [40], see also [41]). Consequently, when viewing normal text, a range of coarse and fine scale visual content from a word is likely to combine to provide normal levels of reading performance. Naturally, therefore, when only coarse or very coarse visual content is present in text (as in the present study), performance will not be as good as normal because the additional input from finer scale visual content that would normally subsequently occur is now prevented. Similarly, when only the fine or very fine visual content of text is present in text, initial lexical activation caused by the coarser visual content that would normally be present in text would not be possible, and so lexical processing would be disrupted. In each case, the loss of the rich visual content normally available in text would impair word recognition, particularly the recognition of less frequent words. This would explain why, consistent with the findings of the present research, restricting the visual content of text generally makes reading more difficult and may sometimes produce larger than normal effects of lexical frequency.

Different types of visual content may also differ in their contribution to processing words at different locations on the page. In particular, while a broad range of visual content contributes to foveal processing, sensitivity to finer visual content outside foveal vision is much reduced [42], [43], and so only more coarse visual content may contribute to processing words outside foveal vision. This includes coarse-scale information about word length and word boundaries that can support eye guidance [28], [44] and information that can help process the identity of the next word before it is fixated [45], [46]. Often this processing is sufficient for identifying short or highly predictable words still in parafoveal vision, and for these words to be skipped so that the eyes move on to the next word in the line of text [35], [36]. However, although there is evidence that readers can identify the first few letters of longer words in parafoveal vision ([47]–[49]; but see [50]), it is unlikely that readers extract details about all the letters in words away from fixation. Consequently, coarse-scale cues to word identities, including information about word length and letter groups, may play an important role in parafoveal processing during reading.

In the present experiment, readers were more likely to show normal rates of skipping target words when text was displayed as very coarse visual content, and only coarse visual content produced effects of word frequency on word-skipping rates that were similar to those obtained for normal displays. This suggests that the coarser visual content from text helps maintain the normal processes of parafoveal word identification that lead to skipping. But when words in the parafovea were fixated, displays containing only coarse or very coarse visual content produced initial fixations that were a little closer than normal to the beginning of words, suggesting that the normal targeting of saccades towards parafoveal words was disrupted. These effects are likely to reflect the different roles of lower spatial frequencies in parafoveal and foveal vision. Since lower spatial frequencies are normally the visual content available for identifying words in parafoveal vision (due to the lower visual acuity of this region), coarse and very coarse visual displays served this purpose well and this explains the more normal processing shown by these displays in skipping rates and the effects of word frequency. This also explains the disruption of these effects for finer visual displays as finer visual content is less visible in the parafovea. However, not all words in the parafovea can be identified sufficiently well for skipping and so, when a parafoveal word needs to be fixated, coarse and very coarse visual content each present foveal processing with an abnormal task because higher spatial frequencies are normally available for foveal word recognition (due to the higher visual acuity of this region). Consequently, when presented with the task of identifying a fixated word using only low spatial frequencies, the solution appears to be to fixate slightly more closely to the beginning of words so that some benefit may be obtained from attending more closely to the cues provided by the beginning letters of these words. Indeed, this explanation resonates with evidence from other studies showing that initial landing positions are closer to word beginnings when the initial letters of words are more difficult to process [51], [52], [53]. In addition, difficulty in identifying fixated words when the spatial frequency content of sentences was filtered may also have contributed to difficulty in processing words in parafoveal vision. It is well-established that parafoveal processing is modulated by foveal processing load, and that difficulty in processing the fixated word limits parafoveal word processing [51], [54]–[58]. Studies that have varied lexical frequency or orthographic regularity of the fixated word have shown no effects of increased foveal load on word-skipping [57], [59], and there is little previous evidence that similar manipulations of foveal load affect initial landing positions in words [60]. However, displaying text in only restricted visual content across the entire visual field (as was done in the present study) is likely to affect both parafoveal and foveal text processing, and this may well have contributed to the observed disruption to normal word-skipping when parafoveal words contained only finer visual content, and to the shift in initial landing positions for foveal words displayed in coarse and very coarse visual content, by both limiting the visibility of parafoveal words and increasing foveal load.

This consideration of the role of spatial frequencies in reading is highly relevant to current models of reading [1], [2], [22], as these models make various crucial assumptions about underlying visual processes. In particular, the E-Z Reader model [1], [2] assumes that a pre-attentive stage of visual processing provides sensory information from the entire page in parallel on each fixation. The nature and precise role of this sensory input has yet to be determined (see [61] for further discussion) but it is assumed it is mediated by visual acuity, and that only foveal vision contains high spatial frequency information that can describe individual letter features. Attention is allocated serially to words in the E-Z Reader model, so that only one word is identified at a time. Therefore, to enable text to be read rapidly, it is assumed that high spatial frequency information about the fixated word provides the input to a first stage of lexical processing that assesses whether the word is likely to be identified imminently, and therefore whether attention can shift to the next word, and the system can begin programming a saccade. By comparison, low spatial frequency information about word length and shape, and the boundaries between words in parafoveal vision, is assumed to provide input to a separate system that selects saccade targets and so determines where the eyes will move next. The underlying assumption of this model, therefore, is that high and low spatial frequencies perform separate and specialized functions during reading. However, there is little evidence from the present research to support this assumption, as our findings show that quite similar eye movement performance is obtained when text is displayed in various forms of restricted visual content, and that a broad range of spatial frequency information can support lexical access and saccade planning during reading. Indeed, our findings suggest that plans about when to move the eyes could be generated using a whole range of visual content from a fixated word. Other models of reading, such as SWIFT [22], make very similar assumptions about visual processes, but differ from the E-Z Reader model primarily by assuming that attention and visual processing is distributed over multiple words, so that adjacent words can be identified in parallel. Unlike the E-Z Reader model, SWIFT does not assume that word identification is the driving force that moves the eyes through text but instead postulates that decisions about when to move the eyes are primarily determined by visual rather than cognitive factors. Nevertheless, it remains an important challenge for both serial and parallel models of reading to explain precisely how the broad range of visual content acquired from text is used to identify words and plan saccades during reading.

In sum, the detailed measures of eye movements used in this study provide fresh insight into the role of visual content during reading. The findings show that various types of visual content can each contribute to eye guidance and lexical processing but no one type of visual content can produce normal eye movement performance. However, a broad range of visual content can activate lexical representations independently during reading. This suggests that while the orchestration of multiple scales of visual content is required for normal eye-guidance during reading, a broad range of visual content can each activate processes of word identification independently, and each may contribute to decisions about when and where to move the eyes during reading.

## Materials and Methods

### Ethics Statement

This research was conducted with the ethical approval of the School of Psychology Ethics Committee at the University of Leicester, and in accordance with the ethical guidelines of the British Psychological Society. All participants gave informed consent in writing.

### Participants

Participants were an opportunity sample of 24 undergraduates from the University of Leicester (mean age = 20 years, range = 18–25 years). All participants were native English speakers, had at least normal acuity, determined by a Bailey-Lovie Eye Chart and an ETDRS Eye Chart, and good contrast-sensitivity, determined by a Hamilton-Veale Eye Chart. Participants reported that they did not suffer from dyslexia or other reading problems.

### Stimuli and Design

Stimuli consisted of 60 matched pairs of sentence frames into which either a high or low frequency word could be inserted as a target word. Sentence frames were matched in each pair and each participant viewed both versions of each sentence, one in each of two different sessions. Each complete sentence was 53 to 67 characters long. Target words were 60 high frequency nouns (mean 249 counts per million according to the CELEX database [62]), and 60 low frequency nouns (mean 2 counts per million) arranged into pairs of high and low frequency words matched for length (mean = 5.32 characters, range = 4–6 characters).

Each sentence was shown in 1 of 6 display conditions, displayed either entirely as normal (unfiltered) or filtered so that only one of 5 types of visual content was present, ranging from very coarse to very fine (see Figure 1). Filtered text was created using MATLAB to digitally filter each sentence into 5 different, 1-octave wide bands of spatial frequencies with peak frequencies of 2.2, 3.5, 4.9, 6.7, 11.1, and 13.7 cycles per degree (cpd) and low-pass and high-pass cut-off frequencies of 1.65–3.3, 2.6–5.2, 5.0–10.0, 8.3–16.6, and 10.3–20.6 cpd (for further details, see [9], [10]). This was achieved by point-wise multiplication in the frequency domain using fourth-order high- and low-pass Butterworth filters. These filters provide a mathematically tractable filter shape that avoids the problems of ringing associated with other filter shapes with a sharp cut-off. The 5 bands of spatial frequencies produced were termed very coarse, course, medium, fine, and very fine, respectively.

A total of 120 sentences were presented to each participant, counterbalanced across participants using the Latin square so that each participant saw 20 sentences (10 containing a high frequency target word and 10 containing a low frequency target word) shown normally and in each of the 5 types of visual content. This ensured that each participant saw an equal number of sentences in each filter condition, and each sentence was seen an equal number of times in each filter condition across participants. Sentences were shown to each participant in randomized order across two sessions, counterbalanced for visual content and target word frequency. Six additional sentences (1 per condition) were presented as practice items at the beginning of each session.

### Apparatus

Eye movements were recorded using an Eyelink 2 K tower-mounted eye-tracker with chin and forehead rest. This eye-tracker has a spatial resolution of .01° and the position of each participant's right eye was sampled at 1000 Hz using corneal reflection and pupil tracking. Sentences were displayed on a 19 inch monitor. At a viewing distance of approximately 85 cm, a 4-letter word subtended approximately 1° (i.e., normal size for reading, [63]).

### Procedure

At the beginning of the experiment, participants were informed that sentences might be difficult to read, and that they should always attempt to read normally, and for comprehension. The eye-tracker was then calibrated. At the start of each trial, a fixation square (equal in size to 1 character space) was presented in the center left of the screen. Once the participant fixated this location accurately, a sentence was presented, with the first letter of the sentence replacing the square. Participants were instructed to press a response key once they finished reading each sentence. Each sentence was then replaced by a comprehension question to which each participant responded. It is common practice to include comprehension questions after sentences to ensure that participants read for comprehension and so a comprehension question was included after each sentence in the present experiment to assess the accuracy of readers' comprehension in the different display conditions. Participants' eye movements for text presented normally were within normal parameters [5] and so there was no indication that including a comprehension question after each sentence affected normal reading performance. Calibration was checked between trials and the eye-tracker was recalibrated as necessary.
